# Relative survival after aortic valve surgery in patients with bicuspid aortic valves

**DOI:** 10.1136/heartjnl-2020-318733

**Published:** 2021-02-23

**Authors:** Natalie Glaser, Veronica Jackson, Per Eriksson, Ulrik Sartipy, Anders Franco-Cereceda

**Affiliations:** 1 Department of Cardiology, Stockholm South General Hospital, Stockholm, Sweden; 2 Department of Molecular Medicine and Surgery, Karolinska Institutet, Stockholm, Sweden; 3 Cardiovascular Medicine Unit, Centre for Molecular Medicine, Department of Medicine Solna, Karolinska Institutet, Karolinska University Hospital, Stockholm, Sweden; 4 Department of Cardiothoracic Surgery, Karolinska University Hospital, Stockholm, Sweden

**Keywords:** epidemiology, aortic valve insufficiency, aortic valve stenosis, congenital abnormalities, heart valve prosthesis

## Abstract

**Objectives:**

The objective of this cohort study was to analyse long-term relative survival in patients with bicuspid aortic valve (BAV) who underwent aortic valve surgery.

**Methods:**

We studied 865 patients with BAVs who participated in three prospective cohort studies of elective, open-heart, aortic valve surgery at the Karolinska University Hospital, Stockholm, Sweden, between 2007 and 2020. The expected survival for the age, sex and calendar year-matched general Swedish population was obtained from the Human Mortality Database. The Ederer II method was used to calculate relative survival, which was used as an estimate of cause-specific survival.

**Results:**

No differences were found in the observed versus expected survival at 1, 5, 10 or 12 years: 99%, 94%, 83% and 76% vs 99%, 93%, 84% and 80%, respectively. The relative survival at 1, 5, 10 and 12 years was 100% (95% CI 99% to 100%), 101% (95% CI 99% to 103%), 99% (95% CI 95% to 103%) and 95% (95% CI 87% to 102%), respectively. The relative survival at the end of follow-up tended to be lower for women than men (86% vs 95%). The mean follow-up was 6.3 years (maximum 13.3 years).

**Conclusions:**

The survival of patients with BAV following aortic valve surgery was excellent and similar to that of the general population. Our results suggest that the timing of surgery according to current guidelines is correct and provide robust long-term survival rates, as well as important information about the natural history of BAV in patients following aortic valve surgery.

## Introduction

Bicuspid aortic valve (BAV) is the most common congenital aortic valve anomaly and affects approximately 1%–2% of the population. Individuals with BAVs have higher risks of valvular dysfunction, endocarditis, and ascending aortic aneurysm and dissection than individuals with tricuspid aortic valves.^1^ Aortic stenosis is the most common clinically relevant consequence of BAV and usually presents between 50 and 70 years of age.^2^ If left untreated, severe aortic valve stenosis is associated with an annual mortality of 25% and the mean duration of survival after diagnosis is 2–3 years.^3^ However, to date, no effective medical treatment for valve disease or aortopathy related to BAV has been identified, although the use of statins and renin-angiotensin-aldosterone system inhibitors has been evaluated.^4 5^ Approximately 50% of patients with BAV require cardiac surgery during their lifetime.^1^ Furthermore, patients with BAV show rapid progression of aortic valve disease and generally require surgery when younger than patients with tricuspid aortic valve disease.^6 7^ The life expectancy of individuals with asymptomatic BAV who are identified in the community is excellent and similar to that of the general population.^7 8^ However, patients who undergo surgical aortic valve replacement (AVR) have a life expectancy that is approximately 2 years shorter than that of the general population.^9^ Furthermore, the prognosis of patients with BAV versus the general population after aortic valve surgery remains to be determined.

To understand the natural history of the condition following aortic valve surgery and to optimise care for patients with BAV, it is important to determine the cause-specific mortality following aortic valve surgery in these patients. Therefore, we performed an observational cohort study to analyse the long-term relative survival of patients with BAV who undergo aortic valve surgery.

## Methods

### Study design

The study database was obtained by combining the data regarding participants with BAVs from three prospective studies: the Advanced Study of Aortic Pathology (ASAP), Mini ASAP (MASAP) and Disease of the Aortic Valve, Ascending Aorta and Coronary Arteries (DAVAACA; online supplemental figure 1). ASAP, MASAP and DAVAACA are prospective, single-centre, cohort studies of patients with aortic valve disease or dilatation of the ascending aorta, or both, who did or did not undergo concomitant coronary artery bypass grafting, and who underwent elective, open-heart, aortic valve surgery at the Department of Cardiothoracic Surgery, Karolinska University Hospital, Stockholm, Sweden, from 2007 onwards. Patients who underwent concomitant replacement of another valve or surgery related to endocarditis were not included. The ASAP study has been described in detail previously,^6^ and the MASAP and DAVAACA studies were follow-on studies of the ASAP study. All the patients gave their written informed consent to participate in these studies.

10.1136/heartjnl-2020-318733.supp1 Supplementary data

BAV morphology was characterised echocardiographically preoperatively and confirmed by the surgeon perioperatively. The BAV phenotype was categorised according to the Sievers and Schmidtke classification.^10^ The baseline characteristics of the patients were obtained from patient-reported charts preoperatively and the Cardiac Surgery Registry, which is a component of the Swedish Web System for Enhancement and Development of Evidence-based Care in Heart Disease Evaluated According to Recommended Therapies registry.^11 12^ The heredity for cardiovascular disease was defined according to the existence of a first-degree relative with myocardial infarction, angina pectoris, transitory ischaemic attack or cerebral vascular lesion of <65 years of age. The continuously updated Total Population Registry maintained by Statistics Sweden was used to obtain the survival status and date of death.^13^ The unique Swedish personal identity number,^14^ which is assigned to all Swedish citizens, was used to cross-link individual patient data between the various registries and the study database.

### Statistical methods

Categorical variables are presented as frequencies and percentages and continuous variables as means and SDs. The outcome measures were all-cause mortality, observed versus expected survival, and relative survival. The all-cause mortality was assessed using the Kaplan-Meier survivor function. The expected survival was estimated using the age, sex and calendar year-matched general Swedish population data that were obtained from the Human Mortality Database (www.mortality.org). The Human Mortality Database contains detailed mortality and population data from 40 countries or areas and is updated continuously. Relative survival is a means of analysing the probability of death resulting from a specific cause that does not require information regarding the cause of death, which is often unreliable or unavailable. In the present study, the relative survival represents the hypothetical scenario in which the only possible cause of death is aortic valve surgery, with or without concomitant thoracic aortic aneurysm surgery or coronary artery bypass grafting, or both, or is related to these. Relative survival is defined as the ratio of the observed, all-cause survival of all the patients to the all-cause survival that would be expected if the patients did not have the disease of interest.^15^ The relative survival and the survival curves were estimated using the *strs* Stata command and the Ederer II method.^15^ The Ederer II method considers the matched individuals from the general population to be at risk until the corresponding patient is censored or dies. An underlying assumption when using relative survival is that the deaths from the specific disease are a negligible proportion of all deaths.^16^ Based on the prevalence of aortic valve disease in the population, it can be assumed that deaths associated with, or due to aortic valve surgery in patients with BAV correspond to a negligible proportion of all deaths. Therefore, the major assumption for using relative survival was considered to be met. Furthermore, the population from the general population used to obtain the expected mortality should be comparable to the patients with the disease under study.^16^ Subgroup analyses were performed according to age group, sex and concomitant procedure. All the patients were followed until the date of death or the end of the follow-up period (25 April 2020). Stata V.16.1 (StataCorp, College Station, Texas) was used for data management and statistical analyses.

### Patient and public involvement

Patients were not involved in the research process of this study.

## Results

A total of 865 patients with BAVs who underwent aortic valve surgery at the Karolinska University Hospital, Stockholm, Sweden, between 2007 and 2020 were included in the study. Their baseline and surgical characteristics are shown in tables 1 and 2, respectively. Their mean age was 60.4 years, 224 (26%) were female, 592 (74%) of the patients had fusion of the left and right cusps (Sievers type 1 left-right) and 603 (71%) underwent surgery because of pure aortic stenosis. Furthermore, 513 (64%) patients received a biological valve prosthesis, 336 (39%) underwent concomitant thoracic aortic aneurysm surgery, 115 (13%) had diabetes and 80 (9.2%) had ischaemic heart disease.

**Table 1:** Baseline characteristics in 865 patients with bicuspid aortic valves who underwent aortic valve surgery in Sweden between 2007 and 2020

	All patients n=865
Age, years, mean (SD)	60.4 (13)
Female sex	224 (26%)
Region of birth	
Non-Nordic countries	69 (8.0%)
Body mass index (kg/cm^2^), mean (SD)	26.7 (4.3)
Cardiovascular heredity	280 (32%)
Smoking	
Never	420 (49%)
Current	75 (8.8%)
Former	359 (42%)
Bicuspid aortic valve phenotype*	
Type 0	66 (8.2%)
Type 1, left-right	592 (74%)
Type 1, right-non	140 (17%)
Type 1, non-left	6 (0.7%)
New York Heart Association classification	
I–II	619 (77%)
III–IV	187 (23%)
Aortic valve pathology	
Aortic stenosis	603 (71%)
Aortic insufficiency	187 (22%)
Combined aortic stenosis/aortic insufficiency	55 (6.5%)
Left ventricular ejection fraction	
>50%	423 (77%)
31%–50%	111 (20%)
<30%	16 (2.9%)
Hypertension	459 (53%)
Diabetes	115 (13%)
Chronic pulmonary disease	57 (6.6%)
Heart failure	65 (7.5%)
Ischaemic heart disease	80 (9.2%)
Atrial fibrillation	55 (6.4%)
Stroke or transitory ischaemic attack	58 (6.7%)
Peripheral arterial disease	55 (6.4%)
Deep venous thrombosis or pulmonary embolism	28 (3.2%)
Prior cardiac surgery	2 (0.2%)
Estimated glomerular filtration rate (mL/min/1.73 m^2^)	
>60	781 (90%)
30–60	78 (9.0%)
<30 or dialysis	6 (0.7%)
Year of surgery	
2007–2010	236 (27%)
2011–2015	372 (43%)
2016–2020	257 (30%)

Data are n (%) unless otherwise noted.

*According to the Sievers and Schmidtke classification.

**Table 2:** Operative characteristics in 865 patients with bicuspid aortic valves who underwent aortic valve surgery in Sweden between 2007 and 2020

	All patients n=865
Aortic valve replacement	798 (92%)
Biological valve prosthesis	513 (64%)
Mechanical valve prosthesis	285 (36%)
Valve sparing procedure	67 (7.8%)
Concomitant thoracic aortic aneurysm surgery	336 (39%)
Concomitant coronary artery bypass grafting	61 (7.1%)

Data are n (%).

### Survival

**Figure 1:**
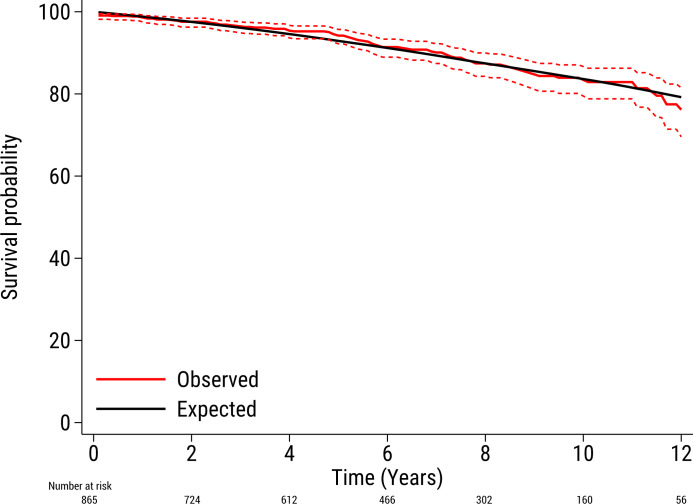
The observed survival (95% CI) in 865 patients with bicuspid aortic valves after aortic valve surgery (red line) and the expected survival of the age, sex and calendar year-matched Swedish population (black line).

**Figure 2:**
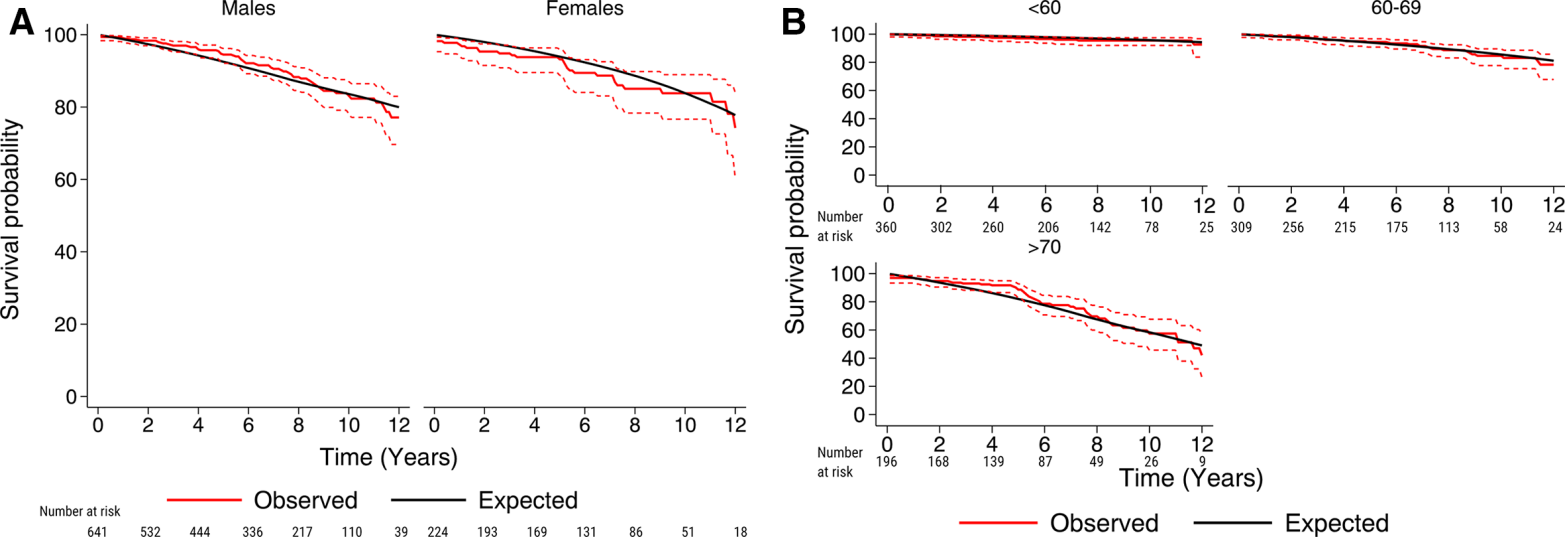
The observed survival (95% CI) in 865 patients with bicuspid aortic valves after aortic valve surgery (red line), according to sex (A) and age categories (B), and the expected survival of the age, sex and calendar year-matched Swedish population (black line).

The mean follow-up period was 6.3 years (maximum 13.3 years) and the total follow-up period was 5460 patient-years. During the study period, 94 (10.9%) patients died. Of these, 7 (0.8%) patients died within the first 30 days following surgery. The survival rates at 1, 5, 10 and 12 years in the full cohort were 99%, 94%, 83% and 76%, respectively. The Kaplan-Meier estimated survival for the full cohort is shown in online supplemental figure 2. The expected survival rates in the Swedish age, sex and calendar year-matched general population at 1, 5, 10 and 12 years were 99%, 93%, 84% and 80%, respectively. The survival rates according to sex and age are shown in table 3. The observed and expected survival curves in the full cohort are shown in figure 1. The observed and expected survival curves, classified according to sex and age, are shown in figure 2A, B.

**Table 3:** Survival by age group and sex in 865 patients with bicuspid aortic valve who underwent aortic valve surgery in Stockholm, Sweden, between 2007 and 2020

	Patients, n (%)	Survival, % (95% CI)		
		**1 year**	**5 years**	**12 years**
Overall	865 (100)	99 (98 to 99)	94 (92 to 96)	76 (70 to 82)
Age group (years)				
<60	360 (42)	99 (98 to 100)	97 (94 to 98)	93 (83 to 97)
60–69	309 (36)	99 (96 to 99)	94 (91 to 97)	78 (68 to 86)
≥70	196 (23)	97 (93 to 99)	89 (83 to 93)	42 (27 to 57)
Sex				
Female	224 (26)	97 (94 to 99)	93 (89 to 96)	75 (61 to 84)
Male	641 (74)	99 (98 to 100)	95 (92 to 96)	77 (70 to 83)

### Relative survival

Relative survival is a way of comparing the survival of individuals with a certain disease with the survival of a reference population who do not have the disease, over a defined follow-up period. The length of the follow-up period depends on the disease under study. Relative survival is calculated as the ratio between the percentage of patients who have the disease and are still alive after a specific period of time and the percentage of people in the general population who are alive at the end of the same time period. The relative survival rate shows if the disease under study shortens life.

The relative survival of the patients at 1, 5, 10 and 12 years in the full study cohort compared with the age, sex and calendar year-matched general population was 100% (95% CI 99% to 100%), 101% (95% CI 99% to 103%), 99% (95% CI 95% to 103%) and 95% (95% CI 87% to 102%), respectively. These results indicate that in the hypothetical scenario where aortic valve surgery or causes related to aortic valve surgery are the only possible causes of death, the survival at 12 years after surgery in patients with BAV would be 95% of the expected survival in the general population. In other words, 12 years after surgery, 5% of the patients would have died due to causes related to having undergone aortic valve surgery. The difference between the relative and the Kaplan-Meier estimated survival at 12 years (95%−80%=15%) represents the deaths due to causes other than those associated with, or due to, aortic valve surgery. In patients who survived the first 30 days following surgery, the corresponding relative survival rates 1, 5, 10 and 12 years after surgery were 101% (95% CI 100% to 101%), 102% (95% CI 100% to 103%), 100% (95% CI 95% to 104%) and 96% (95% CI 88% to 103%), respectively. A non-significant trend was observed towards lower relative survival in women than men at the end of the follow-up period (86% and 95%, respectively). The relative survival of younger patients was similar to that of older patients, and for patients who underwent isolated aortic valve surgery, aortic valve surgery with concomitant thoracic aortic aneurysm surgery and/or coronary artery bypass grafting. The observed, expected and relative survival rates in the full study population, and according to sex and age, are shown in table 4.

**Table 4:** Observed and expected mean survival in patients with bicuspid aortic valve who underwent aortic valve surgery in Stockholm, Sweden, between 2007 and 2020

	Patients (n)	Observed mean survival % (95% CI)	Expected mean survival (%)	Relative survival % (95% CI)
Total study population	865	80 (77 to 84)	87	93 (88 to 97)
At 1 year	807	99 (98 to 99)	99	100 (99 to 100)
At 5 years	539	94 (92 to 96)	93	101 (99 to 103)
At 10 years	159	84 (80 to 87)	84	99 (95 to 103)
At 12 years	55	76 (70 to 82)	80	95 (87 to 102)
Conditional on 30-day survival	858	82 (78 to 85)	87	94 (90 to 98)
At 1 year	807	99 (99 to 100)	99	101 (100 to 101)
At 5 years	539	95 (93 to 96)	93	102 (100 to 103)
At 10 years	159	84 (80 to 87)	84	100 (95 to 104)
At 12 years	55	77 (70 to 82)	80	96 (88 to 103)
Sex				
Female	220	76 (67 to 82)	88	86 (76 to 93)
Male	638	82 (78 to 86)	86	95 (90 to 100)
Age group (years)				
<60	360	94 (89 to 96)	96	97 (92 to 100)
60–69	309	83 (76 to 88)	87	95 (87 to 101)
≥70	196	61 (52 to 69)	68	90 (76 to 102)

## Discussion

The survival of patients with BAV who underwent aortic valve surgery was excellent and similar to that of the general population up to 13 years of follow-up. In these patients, a non-significant trend towards poorer relative survival was found in women compared with men. However, no difference was found in survival compared with the general population in older or younger patients.

Previous studies have shown no difference in survival between young community-dwelling individuals with asymptomatic BAV and the general population, but the risk of cardiac events was higher.^7 8^ Patients with BAV who underwent AVR seem to be at a higher risk of aortic dissection and sudden death than patients with tricuspid aortic valves,^17^ but knowledge regarding the relative survival of older patients with BAV who underwent aortic valve surgery is limited. To provide the best possible care for these patients, it is important to understand the cause-specific mortality after aortic valve surgery. To this end, the present study provides robust observed and relative survival rates in a large, contemporary patient cohort.

Holmgren *et al* analysed observed and relative mortality in patients with bicuspid (n=1131) or tricuspid (n=3782) aortic valves who underwent aortic valve surgery in three Swedish centres between 2005 and 2016.^18^ After a median follow-up of 4.7 years, they found that patients with BAV had a higher long-term survival than patients with transcatheter aortic valve. In line with our results, Holmgren *et al* found that the survival for patients with BAV was similar to the survival in the general population.

McKellar *et al* analysed 1286 patients who underwent AVR for BAV, without surgery of the thoracic aorta, between 1960 and 1995 at the Mayo Clinic, USA.^19^ Their survival rate was similar to the age and sex-matched general population after 5 years, but poorer after 15 years (p<0.001). They also found that female sex was protective in terms of all-cause mortality. However, they included patients who underwent surgery up to six decades ago, which limits the generalisability of their findings to current clinical practice. Furthermore, the perioperative mortality in their study was 2.8%, compared with 0.8% in the present study, which may reflect differences in the patient populations and recent improvements in patient care. In the present analysis, we found no difference in survival between the study population and the general population during 13 years of follow-up. We also found a trend towards lower relative survival in women compared with men. This may be explained by a more adverse risk profile in women undergoing cardiac surgery than in men,^20^ or by effect modification by age in women. Future research focused on sex differences in patients with BAV is needed to further explore this association.

Tzemos *et al* quantified the outcomes of 642 ambulatory adults who were diagnosed with BAV between 1994 and 2001 in Canada.^8^ The mean age of the cohort was 35 years and the 5-year and 10-year mean survival rates were 97% and 96%, respectively. During a mean follow-up period of 9 years, the survival in patients with BAV was similar to that for the age-matched and sex-matched general population. However, 25% of the patients experienced a primary cardiac event (aortic valve or ascending aortic surgery, cardiac death, or hospital admission for heart failure or aortic complication). The 5-year and 10-year survival in the present cohort was lower than that of the Canadian cohort (94% vs 97% and 83% vs 96%, respectively), which can probably be explained by the mean age of the present cohort being 25 years higher. Consistent with the findings of Tzemos *et al*, we found similar survival rates in patients with BAV and the general population. Our results show that patients with BAV have an excellent prognosis, even after aortic valve surgery, and suggest that current guidelines concerning the timing of surgery are appropriate.^21 22^ However, it is likely that the survival rate decreases with time after surgery because the durability of the bioprostheses is limited, and the risk of bleeding that is associated with warfarin treatment and mechanical valves increases with age.^23^ Therefore, further longer term studies of patients with BAV are needed. In line with the results presented by Holmgren *et al*,^18^ we did not find a lower relative survival in patients with coronary artery disease who underwent coronary artery bypass grafting in addition to aortic valve surgery. However, these results have to be interpreted with caution due to the limited number of patients (61 patients or 7.1% of the study cohort). In the present study, 39% of the patients had concomitant replacement of the ascending aorta. Only elective patients were included, and the majority of cases of aortic valve surgery with concomitant ascending aortic surgery are performed electively, which may explain an aggregation of concomitant ascending aortic surgery in our study.

In a previous study, we found a reduction in life expectancy of 1.9 years in patients who underwent AVR compared with the matched general population after approximately 20 years,^9^ and younger patients had a higher reduction in life expectancy than older patients. The greater reduction in life expectancy in younger compared with older patients can probably be explained by the fact that older patients who undergo aortic valve surgery are, in general, healthier than their general population counterparts. In the present study, we found no age-related difference in relative survival. Patients with BAV are in general younger and healthier than patients with tricuspid aortic valves when they undergo cardiac surgery.^6 7^ Lower risks of perioperative and long-term complications associated with aortic valve surgery across all the age groups may explain the similar relative mortalities in the various age groups of patients with BAV.

Previous studies have shown that the BAV phenotype influences the risks of aortic events and significant valve disease in patients with BAV.^24 25^ Furthermore, the left-right bicuspid phenotype is more prevalent in male patients whereas the right-non-phenotype has been linked with ascending aortic dilatation.^26^ Therefore, it is possible that the prognosis after aortic valve surgery differs among patients with different BAV phenotypes and among those with or without aortic ascending aneurysms. Owing to the limited number of patients with true BAV and non-left bicuspid phenotype, this hypothesis could not be tested in the present study.

Transcatheter aortic valve replacement (TAVR) is increasingly used as an alternative to surgical AVR in patients with severe aortic valve disease. Patients with BAV were excluded from the large randomised clinical trials that compared surgical AVR and TAVR,^27 28^ and therefore the treatment strategy for patients with BAV is of great interest. However, observational studies have shown promising results for TAVR in patients with BAV,^29 30^ and therefore the use of TAVR in these patients is expected to increase. The results of our study showed that survival was excellent in patients with BAV after aortic valve surgery. This is important, especially when considering the expected expansion of TAVR in patients with BAV and in patients with low surgical risk.

### Strengths and limitations

The present study provides robust survival rates for a large, contemporary cohort of patients with BAV who underwent aortic valve surgery. However, we did not evaluate outcomes other than survival, such as quality of life, repeat hospitalisation or other late complications following surgery, including bleeding events, thromboembolic complications or prosthetic valve endocarditis.^31^ Because the study design involved intraoperative classification of the valvular anatomy, it is unlikely that patients were misclassified as having BAV. Even though the sample was matched with the general population with regard to age, sex and calendar year, it is possible that other factors, such as comorbidities and socioeconomic status, differed between the BAV cohort and the general population, which must be taken into account when interpreting the results of the study. We believe that the majority of patients with BAV who underwent elective aortic valve surgery were included. However, the capture rate was not 100% because some patients could not be included for various reasons: did not provide consent to participate, logistical reasons (research personnel not available), language barriers, urgent or emergent indication for surgery and other reasons. This possible selection bias may reduce the generalisability of the findings. However, all the patients undergoing elective aortic valve surgery at our institution were screened for eligibility, which increases the external validity of the study. Furthermore, thanks to the high quality of the Swedish national registers, follow-up was complete for the entire cohort.

## Conclusions

The survival rate following aortic valve surgery in patients with BAV was excellent and similar to that of the general population. Our results suggest that surgery at earlier stages of the disease is not likely to be beneficial and that current guidelines concerning the timing of surgery are appropriate. The findings of the present study should facilitate patient education and counselling, as well as clinical decision-making.

**Keymessages**

**What is already known on this subject?**

- The life expectancy of individuals with asymptomatic bicuspid aortic valve who are identified in the community is excellent and similar to that of the general population.

**What might this study add?**

- This study adds information about the prognosis after aortic valve surgery in patients with bicuspid aortic valves versus the general population.

**How might this impact on clinical practice?**

- The findings of the present study could facilitate patient education and counselling as well as clinical decision-making in patients with bicuspid aortic valves who will undergo, or have undergone aortic valve surgery.

## Data Availability

All data relevant to the study are included in the article or uploaded as supplementary information.
