# Supplementary material for: Relative survival after aortic valve surgery in patients with bicuspid aortic valves
Source: Heart. 2021 Feb 23;107(14):1167–72. doi: 10.1136/heartjnl-2020-318733 (PMC8257557; doi:10.1136/heartjnl-2020-318733)

Supplemental Material

Relative survival after aortic valve surgery in patients with bicuspid aortic valves

Natalie Glaser, MD, PhD[1,2], Veronica Jackson, MD, PhD[2], Per Eriksson PhD[3], Ulrik Sartipy, MD, PhD[2,4], Anders Franco-Cereceda, MD, PhD[2,4]

[1]Department of Cardiology, Stockholm South General Hospital, Stockholm, Sweden

[2]Department of Molecular Medicine and Surgery, Karolinska Institutet, Stockholm, Sweden

[3]Cardiovascular Medicine Unit, Centre for Molecular Medicine, Department of Medicine Solna, Karolinska Institutet, Karolinska University Hospital, Stockholm, Sweden

[4]Department of Cardiothoracic Surgery, Karolinska University Hospital, Stockholm, Sweden

Supplemental Figure 1. Flow chart of the study sample.

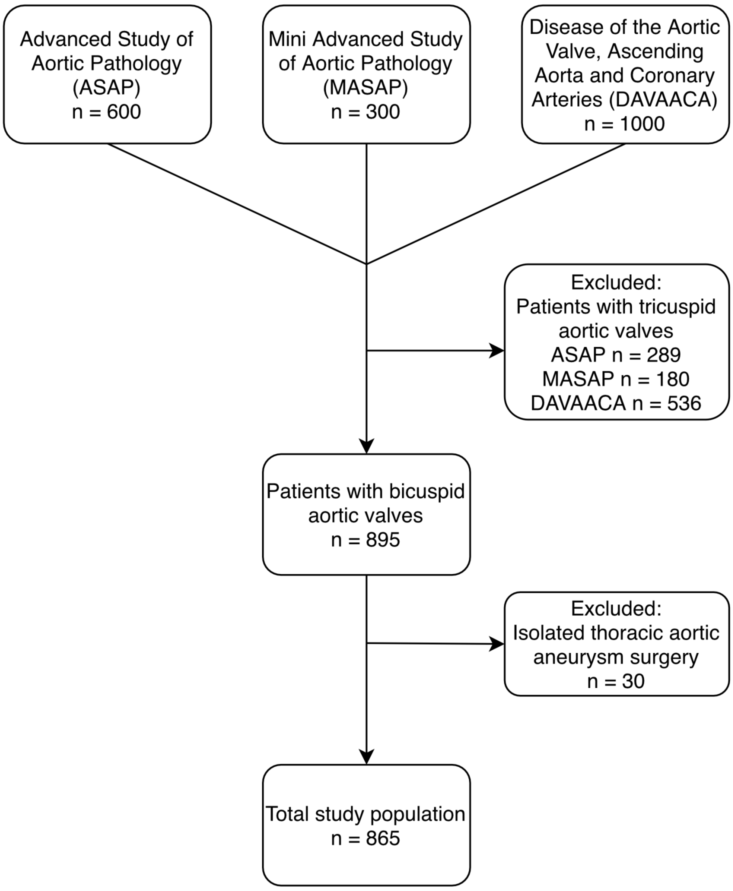

Supplemental Figure 2. Kaplan-Meier-estimated survival in 865 patients with bicuspid aortic valves who underwent aortic valve surgery at the Karolinska University Hospital, Stockholm, Sweden, between 2007 and 2020.

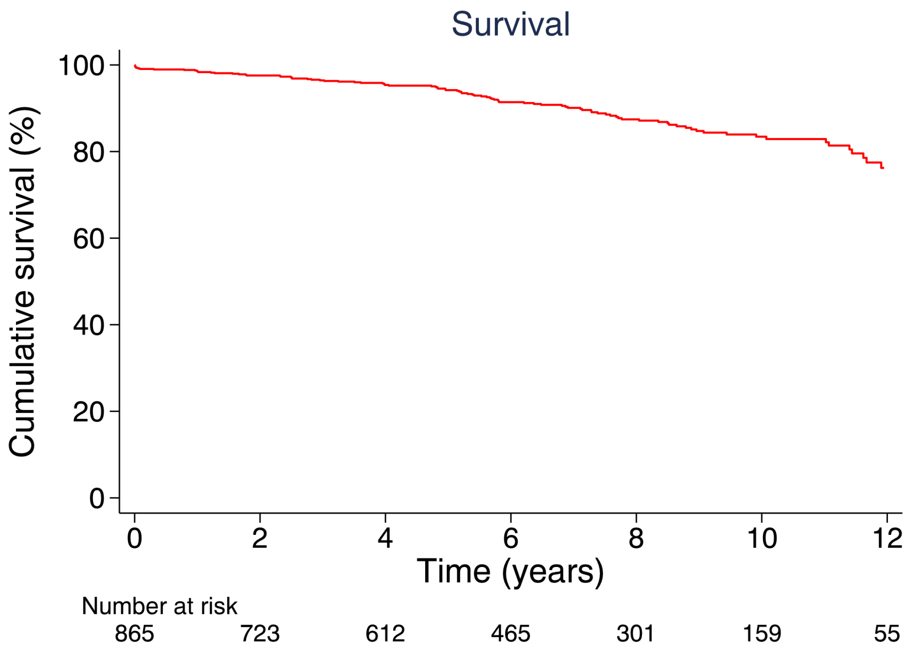

Supplement: Supplementary data [file heartjnl-2020-318733supp001.pdf]
